# The sky is falling

**DOI:** 10.2349/biij.2.3.e29

**Published:** 2006-07-01

**Authors:** BJJ Abdullah, KH Ng

**Affiliations:** Department of Biomedical Imaging, Faculty of Medicine, University of Malaya, Kuala Lumpur, Malaysia

Medical tourism involves travelling to other countries to avail medical, dental, or surgical care. A combination of various factors, such as exorbitant costs of healthcare in industrialised nations, the increased ease and affordability of international travel, favourable currency exchange rates in the global economy, rapidly improving medical technology and standards of care in many countries as well as the ubiquitous Internet, have led to the recent increase in its popularity. The medical tourism market is estimated to grow by USD 2.2 billion with a corresponding increase of USD 60 billion in the healthcare market [1]. Western Europeans and Canadians bypass the long wait periods that are part of their national health plans by getting medical care abroad. Ten per cent of EU patients seek treatment outside their own country and spend an estimated 12 billion Euro [2]. Medical tourism is a rapidly growing industry even in the so-called developing countries, with countries like Mexico, Brazil, Costa Rica, Dominican Republic, Hungary, India, Israel, Jordan, Lithuania, Malaysia, South Africa, Thailand and the Philippines actively promoting it [3].

According to a UN study, the cost differentials for medical services for a variety of procedures may be:

- A heart-valve replacement that would cost USD 200,000 in the US is available for USD 10,000 in India inclusive of the round trip airfare and a vacation package.
- A joint replacement in Thailand with eight days of physical therapy at a luxury resort costs less than USD 9,000.
- Cosmetic-surgery savings are even greater: A full facelift that would cost USD 20,000 in the US is about USD 1,250 in South Africa [1].
- A PET/CT scan performed in Melbourne inclusive of airfare and accommodation in a 4-star hotel is cheaper than what it costs in Singapore, with some pocket money to spare.

Although neither medical city nor medical tourism are new concepts, the scale and volume has exploded beyond expectations. In the last great wave of “globalisation,” in the 18th and 19th century, there were people on the move, seeking out the ultimate cure for various ills not available in the health care system in Britain. What we’re now seeing and experiencing is the development of a second wave of medical tourism [4]. More and more people from all over the world are travelling to other countries not only as tourists who come for sightseeing and shopping but also to get medical, dental, or surgical services from hospitals and other healthcare destinations.

Medical tourists are typically residents of the industrialised nations of the world; the countries they travel to are often less developed with favourable currency exchange ratios. This second global wave of medical tourism is creating new players and companies that are not health specialists, but promoters or brokers between the international patient and the hospital networks. They help arrange surgeries, travel arrangements and tours. However, the Internet also enables increasingly knowledge-rich patients to seek out service providers and make comparisons for themselves, or for specialist firms to expand and include brokerage and specialist services to patients.

Change is always difficult but perpetual and spiralling change is numbing [5]. One would question whose interests have been served by all these advances? This is a complex and emotionally charged issue, with international organisations (WTO), leaders, politicians, economists, lawyers, professional organisations (Royal College of Radiologists, American College of Radiology, etc.), consumers, advocates and antagonists contributing to the debate.

The developments are said to threaten the healthcare system in some of the developed countries and will, if not already, redefine the landscape of medical practice. The clinical procedures that are most heavily promoted in medical tourism and most actively consumed by Westerners are almost all elective in nature and regarded as being the most profitable. This is regardless of where they are provided in Delhi, Denver or Dubai. In purely economic terms, it has been suggested health tourism will therefore result in the increasing outsourcing of the most profitable procedures to offshore suppliers. This carries the fear of a further weakening of the primary-care system in the developed countries, where procedure-based specialties are heavily subsidised. Consequently, fewer resources will be available to aggressively implement the preventive practices to lessen the burden of chronic diseases such as diabetes, heart disease, etc. Medical tourism may also damage the financial and human foundations of the US healthcare system [6] where a less-remunerative career path for medical students may mean that more of the best and brightest will turn away from healthcare careers.

For the developed countries, part of the problem with medical tourism may also be the rate of investment in healthcare information technology. The US has fallen behind other developed countries, as much as a dozen years, in implementing healthcare information technology with little central government involvement [7]. The burgeoning cost of radiology services in the US and the increasing trend towards self-referral also led an editorial [8] to consider the threat of lower-cost overseas teleradiology by advocating both a protectionist approach running in parallel with high-quality medicolegal advice.

Foreign medical graduates and particularly foreign-born nurses play a vital role in the western healthcare system, especially in the USA, Canada, Britain and Australia. As career opportunities in their home countries improve along with the local economies, moving will seem less attractive. Furthermore, although some countries, such as the UK, the US and a few Asian countries, have an overall shortage of consultants in clinical radiology, this position is not reflected throughout the world; some EU countries, India, etc., hold an excess of qualified radiologists. These overseas radiologists, whether working individually or within larger private companies, are ready and available to undertake overseas reporting.

One is now able to buy healthcare services from other countries, at a considerably lower cost. If governments are to stop their citizens from availing this advantage, they must offer sound reasons to do so. The question that must be answered is this: Is healthcare so different that the basic commonsense argument of gain from trade doesn’t hold good here? The inherent inconsistencies, conflicts, discrepancies and inefficiencies of healthcare systems that have evolved over time may no longer financially or economically sustainable. While there are some horror stories and quacks, the data are sparse and the evidence does not support any broad-based significant increase in risk although the legal issues have yet to be resolved.

People, organisations and countries will find a niche where they’re most competitive. No speciality will be spared, from the medical physicists, to bioengineers to surgeons to radiation oncologists, etc. For some countries it will be good quality health care at low cost, for others, it will be the highest quality health care at a higher cost, and for those like Bangkok, Dubai Buenos Aires it will be a geographical advantage. What is happening is the globalisation of trade in health, where individual countries with certain strategic competitive advantages, be it quality, cost or geographical convenience, are exploiting them to the fullest. Health tourism centres/clinics are actively seeking First World “customers” by increasingly pursuing and adopting American and other international best practices to maintain the quality of services.

If medical information, images and diagnosis become mere “commodities” which can be sought, bought, traded and sold over the Internet, then the only real option left is for the medical specialists is to add value [9] by synthesizing all the disparate pieces of information in the context of each individual patient locally, to optimise the selection of treatment and therefore outcomes. Additionally, the doctor must continue to be protective, guiding and showing care for the patient.
